# New York College of Veterinary Surgeons

**Published:** 1897-05

**Authors:** 


### COMMENCEMENT EXERCISES.

### NEW YORK COLLEGE OF VETERINARY SURGEONS.

The commencement exercises of this College were held at the College Hall, 154 East Fifty-seventh Street, April 1st. After the conferring of the oath of Hipprocrates and a short speech by the President of the College, Herman M. Biggs, M.D., the diplomas were awarded to the following gentlemen : James Burns, Newark, N. J.; Fred. L. Bodwell, Manchester, N. H.; William Eckl, Brooklyn, N. Y.; Albert Hecker, Albany, N. Y.; John S. Jones, Poland, N. Y.; John B. Jones, Marlboro, N. J.; Elmer Judkins, Ohioville, N. Y.; Alfred Heard, New York; Alfred J. Pitcher, Warrensburg, N. Y.; Joseph Saile, Brooklyn, N. Y.; Charles F. Spies, Belmar, N. J.; Edward K. Stretch, Hoboken, N. J.; Thomas E. Smith, Jersey City, N. J.; Frank Sturges, Melrose, Mass.; Harry Q. Thompson, Deering, Maine.

Frank P. Sturges was awarded the gold medal for general proficiency, and William M. P. MacKellar the silver medal for passing the best junior examinations. The practical prize—a set of instruments—was awarded to Harry Q. Thompson and Thomas E. Smith.

Later a dinner was served at the Hotel Marlborough, Fred. L. Bodwell, V.S., as toast-master.

Besides the graduating class, the trustees and the Faculty, there were present representatives from the junior classes and a large number of the alumni. The toasts were drunk with enthusiasm, and the responses were attentively heard.

Dr. Hecker said :

It is with feelings of much hesitation that I undertake to express, on behalf of myself and the other members of the class of '97, the appreciation which we all feel for the endeavors which have been made in our behalf by the Faculty of this institution. Neither by words, nor otherwise, is it possible for us to express the worth of your services to us. The measure by which to express the value of services which render a man proficient in a profession by which he is not only to gain his livelihood, but which is to determine his standing in the community where he is to live, and the respect which he is to command from his neighbors, does not exist. It has been your constant aim to do everything in your power to fit us for the life work which we have deliberately selected for ourselves; to perfect us in a profession which, although as a profession comparatively new, has already taken rank as one of the learned professions, which is now receiving the most serious consideration from scientific men, and commands the interest and respect of all humane people. We appreciate the efforts you have made in our behalf. We appreciate that in your work you have been actuated by higher motives than are to be found in any pecuniary consideration. We know that you have felt a sincere and genuine interest in our progress here and in our future, and that not the least of your compensations for your work is to be found in the hope which you entertain that we may succeed in becoming a credit to the college and to our instructors. And if we shall succeed, I know of no one to whom that success will bring more sincere and genuine satisfaction and pleasure than it will to those who have fitted us for our work.

Speaking not only for myself, but in behalf of all those whom I represent, I thank you, and I assure you that we shall all endeavor to be a credit to the profession for which you have assisted us in preparing, and a credit to the college. But we remember, and shall all bear in mind, that "an honest endeavor is worth ten promises."

To the gentlemen of the class of '97, with whom I have been so pleasantly associated, I wish to express my thanks for the honor which they have conferred upon me, and I know of no better way in which I can do so than in the most direct one. Our association here at the college has not only been a pleasant one, but it has, in more ways than one, been a most profitable one. We have not only been able to assist each other to pleasantly while away some leisure hours; we have not only been enabled to be of service to each other in the study of our chosen profession; but the mutual regard and respect which we have learned to entertain for each other have, of necessity, increased in every way our respect for ourselves and our profession.

The remembrances connected with our association here will never cease to be an incentive to us in whatever direction we may turn. This day, to which we have all looked forward with pleasure as the day on which we should be finally launched upon our future work with all its happiness and all its anticipation, would not be complete without its regrets—the regrets of parting from warm friends and pleasant associations. But I hope, and I know, that neither stretch of country nor lapse of years will ever rob us of our mutual friendship and esteem or of the pleasant recollections of these months at the college. I know that any success or good fortune that may come to any one of us will be heard as good news by us all, and that the years cannot move slowly enough to make us forget the pleasures of our association or the regrets of our separation.

Toast, "Professional Ethics," responded to by Prof. R. S. Huidekoper, who said:

You have just finished your course of college education which has prepared you for professional life, and you will now find that the real education which will *fit* you for your professional work begins. Your responsibilities while in college ended with your own individual interests and conception of duty. After answering at roll-call at lecture you could listen for an hour, or take a nap, as you liked; after roll-call in the dissecting-room to pacify me you could work for hours, or, a half hour after my back was turned, I might find you occupied with beer and billiards at Krause's, or talking trotting-horse and stable-business with Kaplan. Now, you are going back to your homes or the location which you have decided upon for your practice, and you will find yourseleves looked upon by very critical eyes. You will find that upon your return to a local community that the public will regard you as a sort of prodigal son, and will not only welcome you, but will give you credit for far more knowledge than you really possess. Your old friends will be seeking knowledge from you, and old horsemen and the fire-fender crank will want to teach you the importance of panaceas which at some time of their lives cured some single case, or they will try to guy and lead you into compromising positions.

In your new life you must determine that you must adopt new friends. To insure your future success you have not only to retain the personal and often selfish friendship of your old acquaintances, but you have to build yourself into the esteem of the strangers whom you will meet. In the first place, be careful of the selection of your office. If you have a stable of your own, fit up a neat room in it near the house; have a neat stall or more for temporary use or keeping a horse, and have your gate and yard in good order. If you have to acquire a location, secure the best you can, from a single office room to a stable, which can be transformed into an infirmary, in the most prominent part of the town which your means will allow. Do not associate yourselves with any livery-stable or hotel, or become identified with them. They will want your services for nothing, and your connection with them will insure you the enmity of others.

Call at once upon the other veterinarians in the locality, and make the acquaintance of the physicians of human medicine and any scientists who are near you. After joining the local or State veterinary society of your region, join if you can the local medical society, for from its meetings you can learn much of the general principles of medicine, and as the members learn to appreciate your special application of medicine they will be of direct use to you in spreading your renown. If there are boards of health or of agriculture within your reach, become associated with them, and obtain a position under them if possible. Even if your remuneration for work for them is not great, you will find that the public gives credit to a man with an official position.

Find at once the average of fees of other veterinarians in your locality, and then determine upon your own, and write out a schedule of them in the front of your ledger, and make them invariable. Some time ago I sent out a circular to all the members of the United States Veterinary Medical Association, and I found that the fees for the larger cities were, on an average, \$2.00 a visit for first animal; varying from \$1.00 to \$3.00. In the country from \$1.00 to \$1.50, and \$2.00. In New England and in localities in the country where the graduates came from Harvard and the University of Pennsylvania, the fees were the highest, and where the graduates were principally from some of the old diploma mills, the fees were the lowest. I found a great want of unanimity in the fees for operations requiring the use of contention, hobbles, etc., and those bringing blood, and as regards the furnishing of medicine.

I would suggest that, according as you find the fees of others in your neighborhood, you adopt a scale somewhat as follows, within reasonable distance of your office: Visit—first animal, \$1.00, \$1.50, or \$2.00; for each additional animal, 50 cents to \$1.00. For minor operations requiring the use of twitch or bringing blood, $3.00 to $5.00; for operations requiring nobbles, $5.00 to $15.00; examination for soundness and consultations, usually, $5.00; for distances beyond a reasonable distance—that is, the radius of your town and its immediate suburbs, an additional charge of 50 cents to $1.00 per mile; for long distances by railroad and for expert services in court or elsewhere requiring an entire day's time, a fee ranging from never less than $10.00, and better $20.00, $25.00, or even $50.00 in special cases, to which you add cost of railroad fare or other transportation. This fee should be equal or greater than the very best day of your practice.

My reason for making the fee for use of contention and minor operations, to bring blood, greater than an ordinary visit, is that you run the risk of injury, the risk of soiling your clothes, and that you have to display expert skill. For use of hobbles the fee should be large, because with all and the best of care your patient will occasionally break its back, and your reputation—through no fault of your own—will in the majority of cases have its back very severely strained, and remain so for some months. This applies also to the operation of castration.

In regard to furnishing medicines, I consider it by far the better plan and preferable to writing prescriptions. Graduate your powders into packages of two for immediate use and repeat, or a dozen for several days; your lotions into pint or quart bottles, and your liquid remedies into bottles containing from a fixed number of doses to the pint to one pint or one quart drenches—one dose to the bottle—and have a fixed price, which is in addition to your visit. Of course, certain special remedies must be a special price.

Having fixed your schedule, adhere to it. You will naturally often be glad to render gratuitous services to the tinker, the peddler, or old Pat, the ashman. But when one liveryman or a decent farmer is paying you for your services, do not let a meaner neighbor persuade you to lessen your charges or bill one cent, under the plea of continuing to employ you, or because he will otherwise employ another veterinarian. Let him employ the cheaper and probably poorer man; he would probably be the last client to pay your diminished bill and the first one to leave you and hunt another cheap man when you have finished your case.

Dress well and keep your clothes in good order; look and act like a gentleman, and your clients will the more cheerfully pay your expenses. Keep out of horse-deals, especially when your professional opinion is in any way involved in them. Treat other veterinarians with courtesy, but in a professional case or consultation do not let any courtesy or feeling of personal friendship bias your judgment or influence your expression of your own views of a case. Marry a pretty girl as soon as you can pay her expenses, and I sincerely hope for you a successful future.

Dr. Sturges responded, saying to the graduates:

These, our last hours together are ones to be long remembered with feelings of both pleasure and regret—pleasure, for the reason that we have reached the goal for which we have been striving for the past three years, and now, that we have attained our object, there is an undercurrent of regret which we cannot suppress, as we leave here, perhaps never to meet again, each man to go his own way, however distant, and to strive by dint of hard work to reach the highest pinnacle of success which is so precious to us all, and which we can only attain by hard and conscientious work.

We possibly had the mistaken idea when first entering upon our studies that to become the so-called "horse-doctor" was an every-day occurrence, but we were soon undeceived, for after attending a few of the preliminary lectures we began to realize that there *was* work, and hard work too; we find in it not only a study to be confined to our three years of college life, but a lifelong one, and one in which to be successful must be grafted with sound common sense.

Why should we not this evening be thankful that we have received our degrees and are entitled to practise our profession, which is a *noble* one if we just stop and consider what we are called upon to do; we are not treating the human being that can tell us its troubles, but dumb animals, which are incapable of articulating a single word, and whose troubles can only be evidenced by signs, which at times are very vague indeed. Our lives now are to be devoted to this. Of course, we are looking for replenishment of our pocket-books; this is a business, and should be conducted as such, but don't let our greed for "that article that glitters" blind us to such an extent that we forget a dumb animal can suffer as does the human being.

Some of us undoubtedly feel despondent when we see the numerous bicycles and horseless carriages, thinking, perhaps, that the horse has had his day, and is to become a thing of the past; but it will never be said of the horse race that "*they* have seen better days." Why were horses given us, and why later become domesticated to answer so many useful as well as pleasurable purposes? To become superseded by those inanimate and shapeless objects—bicycles and motor carriages, which have neither life nor responsive feeling? No; they are here for a purpose, and that purpose is not yet accomplished, nor will it ever be. With all due respect to the brains that invented these mechanical articles which are endeavoring to supersede the horse, but which cannot ever gain the ascendancy over him, we must conclude the bicycle subject.

Now, for a moment stop and think of the numerous openings there are for the veterinarian, such as never before; for instance, that of meat- and milk-inspection; how important this little detail is in itself, when we consider the immense amount of damage that could be done by leaving this branch unguarded; then, again, the veterinarian is called upon for the physical examination of the animals used for vaccine purposes, this vaccine to be used to immune man against that dreaded disease, smallpox, and within the last few years the antitoxins have sprung up and are being extensively used, and we all know, from our short time here, how important they are and to what extent the veterinarian is connected with their production.

We are now professional men, and should conduct ourselves as such, and endeavor to do our work in a professional manner. There are enough empirics at large without any more joining the ranks; become members, active ones, if possible, of the leading veterinary societies, and endeavor to suppress or stamp out this evil, which is degrading our profession; surely, there are enough prominent veterinarians to overcome this, and we must do our share in the matter and not leave it all to the older heads, who, perhaps, have fought until they are weary, and to whom renewed vigor from younger ones would act as a stimulus. It surely is discouraging to a student who, after expending time and money for a collegiate education, to find upon graduating that he has long and hard work in store for him to combat with these so-called "quacks."

We should also make ourselves familiar with both national and local topics, and be able to converse with the people; don't let them get the erroneous idea that our entire knowledge is confined to our profession; get in the best society and hold up our heads. Why should we not? Surely, our aim in life is higher than a great many whose company we may be in at the time.

We have this day taken the Hippocratic oath; let us live up to it.

Now on parting, each and every one of us should make up our minds to uphold the good name the older ones have given the veterinary profession; let each man do his best, and don't have it said that the "Class of '97" sank entirely into oblivion.

To the faculty:

We have now to bid you farewell, you who tried so hard to impart some of your knowledge to us, and which you may be sure we appreciate and shall ever retain in our memory.

We were fortunate in having the opportunity of being students to the able corps of teachers whose signatures we will be only too proud to point to on our diplomas.

We have tried hard to grasp the new ideas advanced us by you, and can understand what a task it must have been, day after day, to endeavor to instil a little of your knowledge into our cloudy brains. We have tried our best to retain it until our poor brains were narcotized with the effort, and is it any surprise, even with the best intentions and respect to you, that we would become drowsy in some of the lectures? Indeed, we have fought many a bitter fight in our heroic endeavors not to look sleepy, and have usually succeeded in winning, perhaps, owing to the hour coming to a close, or to some kindergarten idea which we flattered ourselves we could grasp and retain until our examinations, and then with what joy did we impart it again to you, as does the phonograph to a gaping crowd; but, we all seriously thank you for the foundation of our future life and success; it has been firmly laid, and with this foundation there is no reason why we should not do credit to you who so willingly and freely imparted it to us.

Naturally we are impatient to begin our new life; but withal, we feel deep regret in leaving you, for now, when in doubt, we have no father confessor to come to, no one to lean on, we must rely entirely upon what we have obtained in our brief time as your students, and which cannot but help to pull us through.

There are those among you whose names are known not only here, but across the water, and why should we not feel proud at having served our apprenticeship with you? We hope at some future time to meet you and show our appreciation, other than in words, as these few remarks give but an indefinite idea of what we owe you, and of our thanks. Now, dear teachers, you may rest assured that there is one part of our college life we shall always remember and hold in the highest esteem and love, namely, "The Faculty."

